# What is a compassionate response in the emergency department? Learner evaluation of an End‐of‐Life Essentials online education module

**DOI:** 10.1111/1742-6723.13776

**Published:** 2021-05-05

**Authors:** Deb Rawlings, Megan Winsall, Huahua Yin, Kim Devery

**Affiliations:** ^1^ Palliative and Supportive Services Flinders University Adelaide South Australia Australia

**Keywords:** communication, compassion, emergency department, end‐of‐life care, online learning

## Abstract

**Objective:**

To evaluate the End‐of‐Life Essentials education module ‘Emergency Department End‐of‐Life Care’ and explore learners' views on what constitutes a compassionate response in the ED.

**Methods:**

The present study used a multi‐methods approach. Learners comprised a mix of nurses, doctors and allied health professionals. A quantitative pre‐post evaluation analysis of learners' (*n* = 959) knowledge, skills, attitude and confidence was conducted, along with a qualitative thematic content analysis on learner responses (*n* = 538) to the post‐evaluation question, ‘What is a compassionate response for you in the emergency department?’ Data were extracted for a 12‐month period, 6 May 2019 to 6 May 2020.

**Results:**

Learners' post‐evaluation ranks of knowledge, skill, attitude and confidence were significantly higher than the pre‐evaluation ranks (*P* < 0.001). Emerging themes from the qualitative data were organised into three overarching categories: communication skills (e.g. listening and use of names), care discussion and provision (e.g. provide information and discuss care plans) and humanising healthcare (e.g. emotional support and empathy, taking the time, and offering kindness and comfort).

**Conclusion:**

The ‘Emergency Department End‐of‐Life Care’ module had a significant positive impact on learners in relation to perceived knowledge, skill, attitude and confidence. This evaluation suggests that the End‐of‐Life Essentials ED module could be a useful online learning resource for health professionals.

## Graphical abstract

This paper describes and evaluates an online module that provides end‐of‐life care education relevant to acute hospitals, specific to health professionals working in emergency departments. Apart from a statistically significant positive impact on learners in relation to perceived knowledge, skill, attitude and confidence, important themes emerged: communication skills, care discussion and provision, and humanising healthcare.

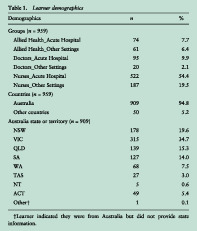

**Key findings**

- End‐of‐life care in an ED is not ideal, with an acute care focus and the view that the ED is not the place for those who are dying.
- The End‐of‐Life Essentials ED education module has enabled learners to identify what compassion in end‐of‐life care looks like, with a focus on communication skills, care discussion and provision.
- It is important to acknowledge that end‐of‐life conversations will continue to be held in ED and that good quality, humanised, responsive care is required.

## Introduction

Around half of expected deaths in Australia occur in hospital settings, with this figure predicted to double in the next 25 years.^1^ Nearly 5000 people died in Australian EDs in 2017–2018,^2^ and while the majority of these will have been unexpected, there will also have been those who died that were at the end‐of‐life,^3^ many of whom will be older people.^4^

If someone at the end‐of‐life does require hospital admission, then they will likely present to the ED, requiring the optimisation of end‐of‐life care.^5^ The reality of a busy ED although is largely focussed on ‘saving’ lives, on curative approaches, and assessment for reversible illness and deterioration.^6^ ED presentations for the patient who is at the end‐of‐life requires a change of focus to incorporate end‐of‐life care;^7^, ^8^ for example, recognising that a patient is dying, incorporation of shared decision‐making and possibly withdrawal of interventions. There is a perception that the ED is not a place for care of the dying;^8^ however, dying occurs regularly in these settings and according to Wang *et al*. ‘EDs are an opportune entry point into the palliative care continuum’.^9^

In 2016, the Australian Government Department of Health funded the End‐of‐Life Essentials (EOLE) project, which aims to provide evidence‐based online education on end‐of‐life care, for doctors, nurses and allied health professionals who work in acute hospitals (https://www.endoflifeessentials.com.au/). EOLE is based on the Australian Commission on Safety and Quality in Health Care National Consensus Statement: essential elements for safe and high‐quality end‐of‐life care.^10^ The project translates the five processes of care elements from the statement: patient centred care; teamwork; goals of care; using triggers; responding to concerns, directly into a suite of 10 education modules each developed following consultation with industry and clinical partners.^11^

‘Emergency Department End‐of‐Life Care’ (hereafter referred to as the ED module) features in the suite of EOLE modules, and includes education around end‐of‐life care in EDs (e.g. delivering compassionate care, having conversations around advanced care planning).^12^

The purpose of the present study was to evaluate the ED module, and to explore learners' views on what constitutes a compassionate response in the ED.

## Methods

### The EOLE modules

The EOLE education modules are freely available online and are designed to build capacity of doctors, nurses and allied health professionals working in acute hospitals in delivering end‐of‐life care. Learners register to access the education, and to date, there have been close to 20 000 registrations, the majority of whom are from Australia, but with many also registered from overseas. There are currently 10 EOLE modules, each with a specific focus on clinical processes of care. Each module consists of online learning content in the form of written information, graphics and videos demonstrating practical care scenarios. There is an accompanying implementation toolkit that provides information and resources as well as a checklist with suggestions for practice.

Targeted to health professionals working in hospital EDs, the ED module has a specific set of learning outcomes, listed below:

- Recognise the importance of assessing the previous 12 months of a patient's life as well as the current episode/presentation of care.
- Define end‐of‐life care in the ED.
- Illustrate the importance of end‐of‐life communications in the ED.
- Appraise the opportunities for end‐of‐life conversations and advanced care planning conducted in a compassionate manner in fast‐paced clinical environments.
- Recognise the range of emotions and responses that are common in end‐of‐life care for staff, family and patients.
- Recognise the opportunities for community liaison about end‐of‐life care and the beginnings of optimal grieving and bereavement.

As part of ongoing quality processes, the ED module was guided by an advisory group who were involved from conceptualisation (including development of learning outcomes) to module delivery. The module was then peer reviewed by doctors and nurses working in EDs across Australia. Upon registration, learners gain access to all 10 modules, and can electively engage with any or all of them. The modules are self‐paced, and learners can take anywhere from 30 min to one and a half hours to complete, depending on how deeply they delve into the learning resources.

### Evaluation framework

The evaluation of all 10 EOLE modules is ongoing and built into the module design via data capture tools in the website. Learners are invited to complete pre‐test/post‐test questions and a free‐text question embedded in each EOLE module. The former are consistent across modules and the latter are relevant to the content of each module. A multi‐methods approach enabled us to complement the quantitative evaluation with qualitative learner descriptions of what they had taken away from the education, adding breadth and depth to the findings.^13^

### Sampling

The participants in this evaluation were learners (healthcare professionals) who had accessed the EOLE website and engaged with the ED module. Learners are self‐selected in that they register on the EOLE website and choose which modules to engage with.

### Data collection

The ED module became available to learners in December 2018. Data (both quantitative and qualitative) were extracted for a 12‐month period, 6 May 2019 to 6 May 2020. For this evaluation, although quantitative and qualitative data were collected on separate platforms, we wanted to extract and analyse data from both for the same timeframe, to ensure consistency. Twelve months was a trade‐off between achieving a large sample size for quantitative analysis, which was representative of the target population, while not having an over‐burdensome amount of qualitative data for the thematic analysis.

#### Quantitative data

The ED module has an embedded evaluation framework, with pre‐test, post‐test data on learners' perceived knowledge, skills, attitude, confidence routinely collected.^11^

The pre‐evaluation questionnaire was set out at the beginning of ED module under the header ‘In thinking about providing end‐of‐life care in the Emergency Department…’. Learners were asked to select ‘strongly disagree’, ‘disagree’, ‘neutral’, ‘agree’, or ‘strongly agree’ in response to the following four statements:

- I have sufficient knowledge in providing end‐of‐life care.
- I am skilled in providing end‐of‐life care.
- I have a positive attitude towards end‐of‐life care.
- I am confident in my ability to provide good end‐of‐life care.

The post‐evaluation questionnaire was set out at the end of ED module. Learners were asked to respond to the four identical statements about end‐of‐life care knowledge, skill, attitude and confidence under the header ‘Since completing this module, in thinking about providing end‐of‐life care in the Emergency Department…’.

Learners who did not provide any responses were excluded from analysis. The pre‐ and post‐evaluation responses were deidentified and imported into SPSS (version 25.00; IBM, Armonk, NY, USA) separately, then merged using the SPSS merge function with the key variable *userID*. In total, data from 959 learners who completed at least one pre‐ or post‐evaluation question were included for quantitative analysis.

#### Qualitative data

Learner statements (one statement per learner) responding to the free‐text response question posed at the end of the module: ‘What is a compassionate response for you in the emergency department’ were extracted from the EOLE learning platform. The data were cleaned, de‐identified, and imported into NVivo 12 software package (QSR International Pty Ltd, Chadstone, VIC, Australia).

### Ethical considerations

Ethics approval was obtained from the Flinders University Human Research Ethics Committee (Project 7012) in relation to overall project evaluation, and includes the pre‐test/post‐test and open‐ended questions and quizzes. Participation is voluntary (opt‐in) with no forced answers. Learners can engage with module content without participating in the evaluation.

### Data analysis

This is an evaluative study with research questions derived from the project education materials,^14^ with a rigorous approach to analysis undertaken to allow generation of knowledge that can lead to practical applications.^15^

#### Quantitative data

Data were analysed using IBM SPSS Statistics version 25. Categorical data were summarised using frequency and percentages. Wilcoxon signed‐rank test was used for comparison of pre‐ and post‐evaluation data. Effect size was calculated based on the method recommended by Fritz *et al*., 0.5 was considered a large effect, 0.3 was considered a medium effect and 0.1 was considered as a small effect.^16^ *P* < 0.05 was considered statistically significant.

#### Qualitative data

Data were analysed using NVivo 12. Pragmatism was used to guide the qualitative analysis, an approach which focusses on using methods that are best suited to answer the specific research question.^17^ Thematic content analysis was conducted to identify key themes emerging from the data, a method chosen because of its suitability for analysing data on multi‐layered healthcare phenomena.^18^ Author MW completed coding for all data and created a coding scheme. An inductive, open approach was used to privilege learner voices and phrasing.^19^ Learner statements were coded line‐by‐line, with conceptual grouping of similar words and sections of text, and codes added as new concepts emerged.^18^, ^19^ Axial coding was then used to organise the codes into overarching categories, and to develop and refine the themes.^19^

To add rigour and improve reliability,^18^ authors MW and DR reviewed and discussed the analysis process and coding schema in detail, with minor modifications made. MW, DR and KD then further discussed and refined the themes. In addressing reflexivity in the data analysis,^20^ it is of note that author MW led the coding from a neutral position with no involvement in module development, and no palliative care background. Data analysis was inductive and based on learners' own words, with all authors conscious of the need for this to drive analysis rather than any pre‐conceptions.

## Results

### Quantitative findings

A total of 1007 learners accessed the pre‐evaluation survey, and 951 answered at least one question, the response rate being 94.4%; 871 learners accessed the post‐evaluation survey, and 821 answered at least one question, the response rate being 94.3%.

#### Demographics

Of the 959 learners, 709 (73.9%) were nurses, 135 (14.1%) were allied health professionals and 115 (12.0%) were doctors. Most learners (72.1%, *n* = 691) were from acute hospital settings (Table 1). Learners from ‘Other settings’ were not asked to specify further on the nature of their professional role or setting. This sample of learners mostly represented the composition and proportion of Australian healthcare workers in public hospitals,^21^ and showed a good geographical spread across states.

**TABLE 1:** Learner demographics

Demographics	*n*	%
Groups (*n* = 959)		
Allied health – acute hospital	74	7.7
Allied health – other settings	61	6.4
Doctors – acute hospital	95	9.9
Doctors – other settings	20	2.1
Nurses – acute hospital	522	54.4
Nurses – other settings	187	19.5
Countries (*n* = 959)		
Australia	909	94.8
Other countries	50	5.2
Australia state or territory (*n* = 909)		
NSW	178	19.6
VIC	315	34.7
QLD	139	15.3
SA	127	14.0
WA	68	7.5
TAS	27	3.0
NT	5	0.6
ACT	49	5.4
Other†	1	0.1

† Learner indicated they were from Australia but did not provide state information.

#### Impact on learners' perceived knowledge, skill, attitude and confidence

Wilcoxon signed‐rank test showed that the post‐evaluation ranks of learners' perceived knowledge, skill, attitude and confidence were statistically significantly higher compared to the pre‐evaluation ranks, the effect size ranged from small to medium (Table 2).

**TABLE 2:** Learners' perceived knowledge, skill, attitude and confidence in providing end‐of‐life care in the ED

Statements	Pre‐evaluation, mean ± SD (*n*)	Post‐evaluation, mean ± SD (*n*)	Wilcoxon (Z) (*n*)	*P*‐value	Effect size
I have sufficient knowledge in providing end‐of‐life care	3.43 ± 0.89 (950)	3.91 ± 0.72 (820)	−15.303 (811)	<0.001	−0.38
I am skilled in providing end‐of‐life care	3.40 ± 0.93 (944)	3.79 ± 0.78 (813)	−13.575 (800)	<0.001	−0.34
I have a positive attitude towards end‐of‐life care	3.97 ± 0.80 (941)	4.25 ± 0.64 (810)	−11.749 (794)	<0.001	−0.29
I am confident in my ability to provide good end‐of‐life care	3.60 ± 0.87 (946)	3.96 ± 0.71 (813)	−13.168 (800)	<0.001	−0.33

Scores reported are average ratings on a 5‐point Likert scale: 1 = strongly disagree; 2 = disagree; 3 = neutral; 4 = agree; 5 = strongly agree. Statistical significance was considered as *P* < 0.05.

### Qualitative findings

A total of 538 learner statements (one statement per learner) responding to the free‐text response question were thematically analysed. The emerging themes were organised into three overarching categories: communication skills, care discussion and provision, and humanising healthcare. Theme descriptions, quantitative counts of codes (number and percentage of learner statements which related to each category and theme) and exemplar learner quotes are presented below (Tables 3, 4, 5).

**TABLE 3:** Descriptions of themes within the category ‘communication skills’ (*n* = 211; 39.2%)

Theme	Description	No. (%) learners	Exemplar quotes
Listening	Some learners described good listening skills as part of a compassionate response, including actively listening, giving patient and family time to talk, allowing for silences, making eye contact and sitting at eye level	132 (24.5%) learner statements related to this theme	‘A compassionate response is a listening ear’ ‘Provided gaps of silence if needed’ ‘Deeply, quietly listening, validating’
Use of names	Some learners discussed the importance of using names in interactions with patients and families, including introducing oneself, and using the names of patients and their family members during conversations	81 (15.1%) learner statements related to this theme	‘A compassionate response for me is introducing myself’ ‘First name basis between patients, family and staff’
Being open, honest, and clear	As part of a compassionate response, some learners described being open to conversation with the patient and their family, being honest about the situation, and ensuring clarity in their use of language, for example ‘no medical jargon’	68 (12.6%) learner statements related to this theme	‘Being completely honest to patients, family or friends about what is happening or what has happened’ ‘Using appropriate language and no medical jargon’

**TABLE 4:** Descriptions of themes within the category ‘care discussion and provision’ (*n* = 250; 46.5%)

Theme	Description	No. (%) learners	Exemplar quotes
Provide information and answer questions	Some learners viewed the provision of accurate information to the patient and family as being part of a compassionate response, this included checking in with patients and family to establish their current level of understanding, educating and providing information about the current situation, prognosis, or treatment and what to expect, and allowing for and answering any questions	143 (26.6%) learner statements related to this theme	‘Checking in with the person to gain a sense of their understanding of the situation’ ‘Being able to inform them of all the information on what to expect from here. Also to be able to inform them correctly of what has been happening since the arrival of their loved one into the emergency department’ ‘Always ask – “Do you have any questions for me?”’
Discuss EOL care plans; incorporate needs, goals, and wishes	Some learners commented on the importance of engaging patients and their families in discussion around care plans, prioritising their needs, goals and wishes, and letting them make choices regarding their own care, that is enable shared decision‐making	101 (18.8%) learner statements related to this theme	‘A compassionate response is one where the ACD is created though patient centred care and family input where applicable’ ‘Understanding/getting to know the patient and their family and the patients wishes, assisting the patient to have comfortable and meaningful care while they are in hospital with their wishes at the forefront of all decisions, having a clear plan and having a clear discussion with the patient or their family about their advanced care plan’
Facilitate connection with supports and services	Some learners commented that part of their compassionate response was to refer patients and families on to appropriate supports and services, for example social worker, pastoral care, funeral planning, as well as offering to call relatives for additional support if needed	52 (9.7%) learner statements related to this theme	‘Advise relatives of services that may be required/available’ ‘Linking the person/family to appropriate services, such as social workers or spiritual guides’ ‘Offer to call someone, give them support contacts if required’
Pain and symptom management	Providing the patient with adequate pain relief and effectively managing their symptoms, was also mentioned by some learners	28 (5.2%) learner statements related to this theme	‘Prompt effective symptom management a priority’

**TABLE 5:** Descriptions of themes within the category ‘humanising healthcare’ (*n* = 382; 71.0%)

Theme	Description	No. (%) learners	Exemplar quotes
Emotional support and empathy	Some learners discussed providing emotional support to patients and families, including acknowledging, validating, and being sensitive to their emotions and grief	180 (33.5%) learner statements related to this theme	‘Acknowledging their feelings and not attempting to fix how they feel’ ‘Showing that I care and want to know and understand their feelings’ ‘The compassionate response in the emergency department for me is to recognise the vulnerability of the people and respond in the empathetic rather than sympathetic way’
Taking the time	Part of a compassionate response to some learners was taking the time to sit with patients and family members, talking with them, adopting an ‘unhurried manner’, and just ‘being there’ to attend their needs	132 (24.5%) learner statements related to this theme	‘Not seeming too busy to be in their presence’ ‘Being present, not just focused on tasks that need to be done’ ‘Sitting with a patient and talking slowly and calmly in all of the chaos’
Time, space and privacy for the family	Part of a compassionate response to some learners was giving the patient's family a sufficient amount of time and privacy when faced with a loved one's death, including providing a private space, and allowing them to be with the dying or deceased patient for as long as they need	84 (15.6%) learner statements related to this theme	‘A place for relatives to go for privacy to discuss the situation and grieve’ ‘Ask if they would like to see the family member who has died, provide a quiet place for this to happen’ ‘Private time with the body to say goodbye’
Offer kindness and comfort	Part of a compassionate response to learners was offering kind and comforting gestures to patients and their families, for example through specific acts of kindness like offering a cup of tea	75 (13.9%) learner statements related to this theme	‘Offering kind gestures like cup of tea’ ‘Providing small comforts, a drink, a rug, a more comfortable chair’ ‘Little acts of kindness go a very long way and decrease stress and anxiety’
Respect for each person	Some learners commented on the importance of catering or tailoring care to meet the needs of each individual person, as well as treating each patient and family member with the utmost respect	51 (9.5%) learner statements related to this theme	‘We need to make the patient feel like a person’ ‘Compassionate response in the emergency department for me means that you are seeing the patient beyond their medical condition. You see them and treat them as a person’ ‘Humanising the experience’
Use of touch	Part of a compassionate response to some learners was the use of touch while caring for a patient or family member, for example through holding their hand or giving them a hug	17 (3.2%) learner statements related to this theme	‘Holding the hand of the patient’ ‘If appropriate I like to squeeze the patients hand while I am listening to them, just to let them know that I hear them and I care’ ‘Being respectful with touching as I do not practice hugging, touching etc. but I will ask permission if I think it may be a compassionate response’
Ensure patient dignity	Some learners commented on the importance of maintaining and upholding a patients' dignity as part of a compassionate care response	14 (2.6%) learner statements related to this theme	‘Maintaining their dignity’ ‘Provide dignified and compassionate care despite the chaotic environment’

## Discussion

Results from the pre‐/post‐evaluation showed that the ED module learning had a significant positive impact on learners' perceived knowledge, skill, attitude and confidence in providing end‐of‐life care in the ED. This suggests that the ED module could be a useful online learning resource for health professionals.

The overarching themes from the present study, wholly derived from the data are: communication skills, care discussion and provision, and humanising healthcare, which incidentally mirror three of the themes derived from interviews with hospital patients on what they view as important (Effective communication and shared decision making, Expert care, Respectful and Compassionate care),^5^ highlighting their relevance and validity in clinical care. By comparing our results to the published literature, we found the experiences of our learners aligned with those of patients.

### Communication skills

Communication is at the forefront of good quality care and never more importantly in end‐of‐life care.^11^ However, there are considerations in settings such as ED, where extra pressures include making way for the next patient,^7^ the lack of time^22^ and specific palliative care training,^23^ all of which impact on care provision.^24^

It is also important to note that healthcare professionals often find end‐of‐life communication confronting and challenging, with over 60% of participants in one study identifying that further training in these aspects of quality end‐of‐life care are required.^25^ Communication skills were found in our study to include: emotional support and empathy, taking the time, listening, using people's names, and being honest, open and clear.

### Care discussion and provision

Care provision in our study includes providing information and answering questions, often as part of discussions around goals of care,^26^ and end‐of‐life plans that ideally incorporate the needs, goals, and wishes of the patient and family. Early end‐of‐life discussions are important and can potentially avert crisis presentations to ED, with Bone *et al*. describing lower attendance at ED when families were aware that the patient was dying.^4^ Communication in ED will often occur in a crisis situation with no previous planning or importance conversations taking place.^22^ In a review by Cooper *et al*., such discussions were found to be more appropriate with clinicians who were familiar with the patient,^7^ which could include primary care teams or the admitting team if the patient has had regular admissions.^24^

Although specialist emergency medicine training in the Australasian context includes education around end‐of‐life care,^27^, ^28^ a lack of specific palliative care content is common in many undergraduate health professional curricula.^29^ Education and training are seen as important to ensure good end‐of‐life care that includes optimal pain and symptom management as well as communication.^7^ Palliative care competencies would help embed end‐of‐life care not only in relation to skills, but as an integral part of the role of both doctors and nurses in the ED.^7^

Jelinek *et al*. reported that waiting times in ED can see insufficient analgesia provided to those with advanced cancer, ‘Some expressed concerns that they had inadequate training in pain management or other specialised areas of symptom control that were necessary to care for these patients’.^22^ Gerace *et al*. in their study with ED nurses, found not only a need for adequate pain control, but also a move away from lifesaving procedures.^3^ Access to services can also be a reason for ED attendance and was identified as a theme in our study, ‘facilitate connection with supports and services’, described elsewhere as a failure of long‐term care,^7^ with ED presentation mentioned as a last resort for some.

### Humanising healthcare

Over two decades ago, Rosenzweig wrote about humanism in the context of ED, ‘defined by identifying the dehumanising aspects of sudden illness and exploring of ways for sustaining the humanity of emergency departments’.^30^ This should be viewed through the palliative care lens of counteracting the biomedical approach with a compassionate one. In our study, this becomes offering kindness and comfort, respecting each individual, using touch and ensuring dignity. It includes simple acts of kindness often forgotten, such as making a cup of tea. It also includes time, space and privacy for the family which affects the quality of care provided,^7^ and which these authors highlight as tensions for healthcare professionals, wanting to prioritise these concerns but unable to do so within time and resource constraints. The ED was generally considered inappropriate for opimal end‐of‐life care, although clinicians did want to provide best practice care.^7^

Humanising healthcare in ED is of more importance when recognising the less than ideal environment for end‐of‐life care.^24^ One study described the ED in relation to dignified end‐of‐life care as ‘hostile’, signifying that steps are required to address the way in which care is provided in this setting.^31^

Busch *et al*. in their systematic review, highlighted barriers to the implementation of humanisation, including traditional medical education models, hospital hierarchies and lack of managerial support.^32^ Youngson and Blennerhassett go further in describing inhumanity as a system failure.^33^ The importance of humanising healthcare is also reflected in the communication themes, with many comments there also resonating within tenets of person‐centred care, particularly important in a busy ED where end‐of‐life care needs may not be prioritised.^34^

### Limitations

There are limitations in that learners are self‐selecting into the EOLE education and then into the ED module, so by default will differ in their participation and responses from those who elect not to do this (e.g. a tendency towards acquiescence).^35^ The self‐report nature of the evaluation did not allow us to ascertain if knowledge improvement or behaviour change did indeed take place, with self‐assessment not always correlating with objective measures,^36^ although a longitudinal study is planned.

The learner responses to the open‐ended question varied in length (e.g. from one or two words, to multiple paragraphs), and learners were not probed further, potentially reducing the richness of the qualitative data. Qualitative and quantitative data were collected in two different platforms and as such cannot be linked. This is a limitation in that linking these data may have provided a more comprehensive picture of learners' experiences.^6^ A further limitation is that more detailed demographics data were not captured (e.g. role, length of service), thereby limiting a more in‐depth analysis of learner characteristics.

## Conclusion

Provision of end‐of‐life care in a busy ED is not ideal, with time and space issues as well as competing demands, an acute curative care focus, and the view that the ED is not the place for those who are dying. As a response to the EOLE ED education module, learners have identified what compassion in end‐of‐life care looks like, and have described it in terms of communication skills, care discussion and provision, and humanising healthcare. In recognition that goals of care and planning conversations should occur with those healthcare professionals who usually look after them, learners also understand that these conversations will continue to be held in ED and that good quality, humanised, responsive care is required.

## Data Availability

The data that support the findings of this study are available on request from the corresponding author. The data are not publicly available due to privacy or ethical restrictions.
